# Age-Specific Characteristics and Malignancy Risk of Ovarian Teratomas: A Retrospective Single-Centre Study

**DOI:** 10.3390/jcm14165872

**Published:** 2025-08-20

**Authors:** Su Hyeon Choi, Haeng Jun Jeon, Bohye Gil, Seyeon Won, Nara Lee, Sohyun Shim, Mi Kyoung Kim, Yong Wook Jung, Seok Ju Seong, Mi-La Kim

**Affiliations:** 1 Department of Obstetrics and Gynecology, CHA Gangnam Medical Center, CHA University School of Medicine, 566 Nonhyeon-ro, Gangnam-gu, Seoul 06135, Republic of Korea; k345@chamc.co.kr (S.H.C.); b223022@chamc.co.kr (B.G.); drtong85@chamc.co.kr (S.W.); naradd@chamc.co.kr (N.L.); simuso@chamc.co.kr (S.S.); ra13811@chamc.co.kr (M.K.K.); dumbung@chamc.co.kr (Y.W.J.); sjseongcheil@naver.com (S.J.S.); 2 Department of Obstetrics and Gynecology, Fertility Center of CHA Gangnam Medical Center, CHA University School of Medicine, Seoul 06125, Republic of Korea; aln6012@chamc.co.kr

**Keywords:** ovarian teratoma, malignancy-associated ovarian teratoma, immature teratoma, malignant transformation, age

## Abstract

**Aim:** The aim of this study was to determine the age-specific characteristics of ovarian teratoma and associated malignancies. **Methods:** This retrospective single-centre cohort study included 2181 women with ovarian teratoma who underwent surgery at our institution between January 2008 and April 2019. Malignancies associated with ovarian teratoma were divided into immature teratoma, combined ovarian malignancy, and malignant transformation of mature cystic teratoma. The median patient age was 30 years (range, 7–82) and the median follow-up duration was 10 months (range, 0–152). **Results:** Most ovarian teratomas were detected incidentally, except in patients with abdominal pain under 20 years of age; torsion was significantly more common in this age group (*p* < 0.001). Tumours were larger in the younger age group (*p* < 0.01). The incidence of immature teratoma was 0.5% (*n* = 11), that of combined ovarian malignancy was 0.4% (*n* = 9), and that of malignant transformation was 0.4% (*n* = 9). The median patient age was 24.0 years for immature teratoma and 27.0 years for combined ovarian malignancy. The most common cell type was mucinous borderline tumour (55.6%, *n* = 5). The median patient age of malignant transformation was 33.0 years, and the most common cell type was carcinoid tumour (77.8%, *n* = 7). At our institution, the clinical manifestations of ovarian teratoma varied according to age group, with younger patients being more likely to be symptomatic and to have larger tumours and bilateral tumours. Although there was no statistically significant relationship between age and associated malignancy (*p* = 0.442), most of the malignancies associated with ovarian teratoma were found in childbearing age, not in older age. **Conclusions:** Given the possible associated malignancy with ovarian teratoma, surgeons should perform detailed preoperative evaluations, avoid intraoperative spillage, and perform intraoperative frozen biopsy when appropriate.

## 1. Introduction

Ovarian teratomas include benign mature cystic teratoma (MCT), immature teratoma, and monodermal teratoma (e.g., struma ovarii, carcinoid tumour, and neural tumour) [1]. MCT is the most common ovarian germ cell neoplasm and accounts for 10–20% of all ovarian masses [2,3,4]. More than 80% of MCTs occur during the reproductive years [5] and are composed of various tissues derived from one or more germ cell layers. Ectodermal derivatives are the most common and include the keratinising epidermis, sebaceous and sweat glands, hair follicles, and neuroectodermal tissues. Mesodermal derivatives include muscle, bone, cartilage, fat, and occasionally teeth. Endodermal derivatives include the thyroid and salivary glands as well as respiratory and gastrointestinal tissues [6]. Immature teratomas also contain elements derived from all three germ cell layers; however, these malignant tumours contain immature neuroepithelium and usually affect young women [7]. Although MCTs are prevalent and usually benign, mature teratomas can be associated with malignancy, which may manifest as combined ovarian malignancy (including epithelial ovarian malignancies and borderline tumours) or result from malignant transformation.

Given the benign nature of MCT, surgery may be deferred, especially in younger patients of reproductive age [8]. Therefore, early detection of malignant transformation or malignancy associated with MCT or immature teratoma is important. However, unlike immature teratoma and malignancy associated with MCT, preoperative imaging and biochemical findings are neither specific nor sensitive, intraoperative diagnosis of malignant transformation of MCT is difficult, and guidance for optimal management is not well established owing to the rarity of these tumours [9].

Most of the previous studies on ovarian teratoma have focused on its clinical characteristics in younger women and associated malignancies in older women, and there is a paucity of large-scale analyses evaluating all malignancy subtypes across age group. Therefore, in this study, we reviewed our experience of cases of ovarian teratoma treated at our institution to determine the age-specific characteristics of this tumour and features of immature teratoma, combined ovarian malignancy, and malignant transformation of MCT.

## 2. Methods

### 2.1. Ethics Approval

The study protocol was approved by the Institutional Review Board on the CHA Gangnam Medical Center (GCI-2021-09-001). All processes conducted in this study involving human participants were in accordance with the ethical standards of the institutional committee and with the 1964 Helsinki declaration and its later amendments or comparable ethical standards. Informed consent was not sought, as a retrospective study design was used. Data were anonymized and de-identified before analysis, and, therefore, informed consent was not required.

### 2.2. Patient Population and Data Collection

**Figure 1:**
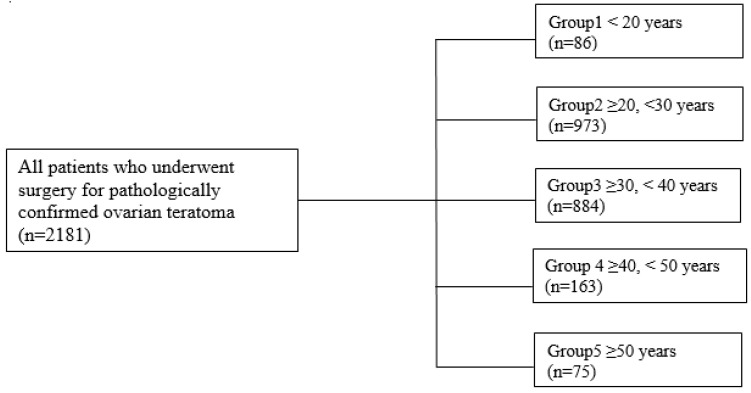
Flow diagram of patient selection process.

We retrospectively reviewed data from 2181 patients who underwent surgery for pathologically confirmed ovarian teratoma at our institution between January 2008 and April 2019 (Figure 1). Malignancies associated with ovarian teratoma were divided into immature teratoma, combined ovarian malignancy, and malignant transformation of MCT. Immature teratomas are distinguished from mature teratomas by the presence of immature neuroepithelium [7,10]. Combined ovarian malignancy included malignant tumour and borderline malignancy as well as both ipsilateral and contralateral ovarian tumours. Malignant transformation included any malignancy arising from an ovarian teratoma [11]. Intraoperative frozen pathology was selectively performed when malignancy was suspected based on preoperative imaging or intraoperative gross findings, or when diagnostic uncertainty could affect surgical decision-making. Data including age at surgery, parity, body mass index, size of the teratoma (for multiple cysts, the diameter of the largest cyst was recorded; if bilateral, the sum of the cyst with the largest diameter on each side was recorded), preoperative cancer antigen 125 (CA125) and cancer antigen 19-9 (CA19-9) levels, previous surgery for ovarian teratoma, preoperative symptoms, location of the ovarian cyst(s), ovarian torsion, associated other ovarian pathology, and postoperative follow-up duration were collected from medical charts. Computed tomography (CT) was the primary imaging modality for preoperative evaluation owing to its broad availability, rapid acquisition, lower cost, and ability to assess both pelvic and abdominal organs in a single scan. Magnetic resonance imaging (MRI) was reserved for cases with inconclusive CT findings or when detailed soft tissue characterization was required. Malignancy suspicion on CT or MRI was based on the presence of solid components, irregular septations, papillary projections, thickened cyst walls, or radiologic evidence of extra-ovarian spread. All imaging studies were reviewed by at least two board-certified gynecologic oncologists, and all pathological diagnoses were confirmed by experienced gynecologic pathologists at our institution to ensure consistency of interpretation. Patients were categorized according to age (<20 years, n = 86; 20–29 years, n = 973; 30–39 years, n = 884; 40–49 years, n = 163; ≥50 years, n = 75).

### 2.3. Statistical Analysis

Categorical variables were compared using Fisher’s exact test or the chi-squared test. Quantitative variables were compared using the Mann–Whitney U test or Kruskal–Wallis test. All statistical analyses were performed using SPSS software (version 25.0; IBM Corp., Armonk, NY, USA). A *p*-value < 0.05 was considered statistically significant.

### 2.4. Data Availability

Data will be available upon reasonable request from the corresponding author. However, the data cannot be made public to maintain women’s privacy and legal reasons, as it contains private health information along with identifiers.

## 3. Results

### 3.1. Age Distribution of Baseline Characteristics

The median age of the 2181 study participants was 30 years (range, 7–82). The median follow-up duration was 10 months (range, 0–154). Table 1 shows the distribution of baseline characteristics according to age. Most ovarian teratomas were detected incidentally, except in patients aged <20 years; 76.7% of these patients had symptoms, most commonly abdominal pain (45.3%). However, there were no symptoms in 62.8% of patients in their 20s, 73.1% of those in their 30s, 69.9% of those in their 40s, and 73.3% of those aged 50 years or older. Abdominal pain was the most common symptom in all age groups. The torsion rate was highest in patients in their 20s (*p* < 0.001). The relationship between mean tumour size and torsion rate was not significant (7.5 ± 3.7 cm without torsion vs. 8.3 ± 3.8 cm with torsion, *p* = 0.133, Mann–Whitney U test). Tumour size varied significantly according to age group (*p* < 0.001) and was largest in younger patients. Bilaterality was more common in the younger age groups (*p* < 0.001).

There were significant differences in serum CA 125 and CA 19-9 levels according to age group. (*p* = 0.026 and *p* < 0.001, respectively, Kruskal–Wallis test). At our institution, the normal range for CA125 is 0–35 U/mL and that for CA19-9 is 0–27 U/mL. In total, 226 (13.4%) of the 1686 patients with CA125 levels available and 347 (42.0%) of 827 patients with CA19-9 levels available had an elevated value. There was no significant relationship between having a CA125 level above the normal range and age (*p* = 0.154); however, an elevated CA19-9 level was significantly more common in younger patients (*p* < 0.001). The serum CA19-9 level correlated with tumour size (normal range vs. elevated CA 19-9 level, 7.3 ± 3.4 cm vs. 9.3 ± 4.3 cm, *p* < 0.001, Mann–Whitney U test) and bilaterality (12.6% vs. 21.2%, *p* = 0.001, chi-squared test) but not with symptom status (35.6% vs. 34.0%, *p* = 0.643, chi-squared test).

### 3.2. Associated Benign Ovarian Tumours with Teratoma

Table 2 shows that 4.8% of all ovarian teratomas were associated with benign ovarian tumour and that the rate increased with advancing age. Ovarian endometrioma was the type most commonly observed.

### 3.3. Malignancy-Associated Cystic Teratoma

As shown in Table 3, the incidence of immature teratoma was 0.5% (11/2181), while the incidence of combined ovarian malignancy and that of malignant transformation were both 0.4% (9/2181). The peak incidence of immature teratoma was in the 20s. The most common type of combined ovarian malignancy was mucinous borderline tumour (55.6%, 5/9) and all combined ovarian malignancies were found in patients under 40 years of age. The most common type of malignant transformation was carcinoid tumour (77.8%, 7/9), which had a peak incidence in the 30s. However, the relationship between malignancy-associated ovarian teratoma and age was not significant (*p* = 0.442, chi-squared test).

MCT and the subcategories of malignancy associated with ovarian teratoma are shown in Table 4. Patients with immature teratoma were significantly younger (*p* = 0.05), were more symptomatic (*p* < 0.001), and had larger tumours (*p* < 0.001) and higher CA125 levels (*p* = 0.002) than those with MCT. However, preoperative CT and MRI raised suspicion for malignancy in only 4 of 11 patients; 8 of these 11 patients underwent intraoperative frozen biopsy and 5 were found to have an immature teratoma. There was no statistically significant relationship between combined malignancy and age, initial symptoms, torsion rate, tumour size, or CA125 level. Preoperative imaging was performed in four of nine patients, and only one was suspected to have malignancy. Intraoperative frozen biopsy was performed in seven patients, six of whom were confirmed to have combined malignancy intraoperatively. Patients with malignant transformation were significantly older than those with MCT (*p* = 0.046); however, there was no significant between-group difference in initial symptoms, torsion rate, tumour size, or CA125 level. Preoperative computed tomography CT or MRI was performed in three patients and did not raise suspicion for malignancy. Only one woman underwent intraoperative frozen biopsy, which confirmed malignant transformation.

Supplementary Table S1 shows the clinical characteristics of 29 patients with malignancy-associated ovarian teratomas. Most patients had early-stage disease, and only one was postmenopausal. Among the 25 patients with available follow-up (median 43 months (range, 1–117)). Only two patients had stage IIB disease, and most had stage I or incomplete staging. During follow-up, three patients experienced recurrence, and seven achieved pregnancy after treatment, all of which resulted in successful deliveries except for one patient lost to follow-up after conception.

## 4. Discussion

In this study, the most common clinical characteristics of ovarian teratoma were abdominal pain, a higher torsion rate, a larger tumour, and a higher rate of bilateral tumours in the younger age groups. The incidence of associated benign ovarian tumours increased with age, the most commonly observed being ovarian endometrioma. There was no significant difference in malignancy-associated ovarian teratoma according to age.

In age-focused analysis of malignancy-associated cystic teratoma, immature teratoma occurred only in the younger age groups and not after the age of 40 years, as reported previously [11]. The median age was 24 years (range, 15–35).

Combined ovarian malignancy occurred at a relatively young age compared with previous studies. The median age of patients with combined malignancy and teratoma was 27 years (range, 16–38) and the most common type was mucinous borderline tumour (62.5%, 5/8; 0.2% of all cases). Mucinous borderline tumour has a reported prevalence of 0.5% and requires careful staging and clinical follow-up [12].

Malignant transformation is generally associated with increasing age; however, in our study, the median age of patients with malignant transformation was 33 years (range, 28–69), and only one patient was postmenopausal. The possibility of malignant transformation is very low at 0.17–2% [13]. In keeping with the predominance of ectodermal derivatives, more than 75–90% of cases of malignant transformation of teratoma are squamous cell carcinoma (SCC), especially in postmenopausal patients [14,15,16,17]. Duffy et al. investigated 1082 MCTs and found that patients over 40 years of age had a higher rate of malignant transformation than their younger counterparts (2.8% vs. 1.5%) [12]. Furthermore, a review of 14 studies found that 12 reported a median age older than 40 years and two studies reported a median age of 36 years (range, 24–70) in 753 patients and 36 years in 1082 patients [12]. However, in our study, the incidence of malignant transformation was 0.4% for all ovarian MCTs. Carcinoid tumour was the most common histological type (77.8%). MCT into carcinoid tumour is extremely rare, and most of these cases have been published as case reports. Recently, Gadducci et al. reported case series of carcinoid tumour in six patients, all of which were confirmed to be stage 1; one died of carcinoid heart disease and the remaining five remained disease-free during a median follow-up of 168 months [18]. The median age of our patients with carcinoid tumour was only 32 years (range, 29–69). Given that our institution specializes in infertility, most gynecological surgeries focus on preserving fertility. For this reason, as well as the very small size of the carcinoid tumours, women under 40 years of age (n = 5) were treated by ovarian cystectomy only and those over this age (n = 2) were treated by unilateral salpingo-oophorectomy. Excluding two patients who were transferred immediately, no evidence of disease was observed in any patient during a median 39 months of follow-up. Three women conceived (one naturally and two by in vitro fertilization) and delivered successfully. However, a larger study is needed to determine the relationship between MCT and carcinoid tumours in young age women.

The younger median age observed in our malignant transformation cases compared with previous reports may reflect referral patterns to our fertility-specialized centre, which treats a high proportion of reproductive-aged women. Sociodemographic factors, such as delayed childbearing and healthcare-seeking behaviour, may also contribute to this finding. This highlights the need for individualized surgical planning that balances oncologic safety with fertility preservation and for tailoring follow-up intensity according to patient age and tumour risk profile.

The reported incidence of SCCs in malignant transformation ranges from 75% to 90% [19,20,21], but in this study was only 11.1% (in one of nine cases). Unlike in the previous reports, in which SCCs usually occurred in older or postmenopausal women, one of our cases was 33 years of age. This patient had stage IC2 disease and underwent unilateral salpingo-oophorectomy with infracolic omentectomy and appendectomy via a laparoscopic approach followed by chemotherapy, which included intraperitoneal cisplatin and six cycles of paclitaxel and carboplatin. She conceived spontaneously, delivered full-term at 21 months postoperatively, and was still alive at 79 months (Supplementary Table S1). Araujo et al. also reported malignant transformation of MCT into SCC in women in Brazil at a median age of 37 years [22]. It has been suggested that there is a long interval between occurrence of MCT and malignant transformation, the pathogenesis of which may involve prolonged exposure to various carcinogens in the pelvic cavity [23]. Araujo et al. proposed human papillomavirus infection as a potential risk factor for malignant transformation of MCT to SCC; however, molecular studies are needed to clarify this suggestion [22].

Additional CT or MRI studies are performed at our centre when the tumour marker level is high or initial ultrasonographic findings are suspicious for malignancy. If malignancy is suspected from preoperative imaging studies or during surgery, an intraoperative frozen biopsy is performed. In this study, based on CT or MRI findings, malignancy was suspected more often in patients with immature teratoma than in those with MCT (36.4% vs. 6.8%, *p* < 0.001). Intraoperative frozen biopsy was performed in 116 patients (5.3%), and malignancy was confirmed by frozen biopsy in 12 (75%) of 16 cases. Therefore, care should be taken to reduce the risk of intraoperative spillage, especially in younger women. Oophorectomy with intraoperative frozen biopsy should be considered in high-risk patients, including women aged over 40 years and those who are postmenopausal.

One of the major concerns with enucleation of a cyst is the decline in ovarian reserve; a certain amount of ovarian tissue is removed, and the remaining ovarian tissue is damaged by coagulation maneuvers. Therefore, surgery is often postponed after a clinical diagnosis of MCT, especially in younger women. Regular ultrasonographic follow-up should be considered in these women to prevent and detect malignant transformation. In clinical practice, these observations emphasize that surgical decision-making should incorporate both age-specific malignancy risk and reproductive goals. In younger patients desiring fertility, ovarian tissue preservation should be maximized, whereas women at higher risk of malignant transformation may benefit from earlier surgical intervention and closer surveillance. In our study, seven patients with malignancy-associated ovarian teratoma conceived after surgery, with six successful deliveries.

This study had some limitations. First, although it is one of the largest studies of ovarian teratoma, the number of patients with associated malignancy was too small to draw a firm conclusion to relationship between MCT and carcinoid tumours in young age women. Therefore, our findings should be interpreted with caution and need confirmation in larger studies. Second, the study had a retrospective design and patient numbers in the different age groups were not evenly distributed. Our institution specializes in infertility, so most of the patients were of reproductive age; therefore, it was not possible to analyze the general characteristics of women of all ages. This single-centre, fertility-focused setting may have influenced both the age distribution, and the spectrum of malignancy observed, introducing potential selection bias. In addition, because our cohort consisted exclusively of Korean women, geographical and ethnic factors may have influenced the incidence, histologic subtypes, and clinical characteristics of malignancy associated with ovarian teratomas. We have therefore emphasized the need for multicenter and multiethnic studies to confirm and generalize our findings. However, this shortcoming may be offset by the fact that the study included many women of reproductive age, who have a higher incidence of teratoma. This study also has the strengths of a relatively large sample size and inclusion of data on pregnancy subsequent to treatment of malignancy-associated ovarian teratoma, and suggests that the most common type of malignant transformation is SCC, albeit not in young women.

In conclusion, the findings of this study suggest that the clinical manifestations of ovarian teratoma vary according to patient age, in that younger patients are more likely to have symptoms, a larger tumour size, and bilateral tumours. Most of the malignancies associated with ovarian teratoma were found in women of childbearing age, although there was no significant difference in frequency according to age group. Therefore, considering the possibility of malignancy associated with ovarian teratoma, surgeons should take care to conduct appropriate preoperative evaluations, minimize the risk of intraoperative spillage, and perform intraoperative frozen biopsy as appropriate. Larger multicentre, multiethnic studies are needed to confirm these findings and define the role of molecular profiling in managing rare carcinoid tumours arising from MCT.

## Figures and Tables

**Table 1:** Age distribution of baseline characteristics in teratoma patients.

Age Group	All	Group 1 (<20 Years)	Group 2 (20–30 Years)	Group 3 (30–40 Years)	Group 4 (40–50 Years)	Group 5 (≥50 Years)	*p*-Value
Number of patients	2181	86	973	884	163	75	
Initial symptom, *n* (%)							0.000 ^a^
Irregular mens	106 (4.9%)	15 (17.4%)	58 (6.0%)	30 (3.4%)	3 (1.8%)	0 (0%)	
Abnormal bleeding	139 (6.4%)	6 (7.0%)	62 (6.4%)	55 (6.2%)	13 (8.0%)	3 (4.0%)	
Abdominal pain	433 (19.9%)	39 (45.3%)	217 (22.3%)	135 (15.3%)	32 (19.6%)	10 (13.3%)	
Palpable mass or abdominal distension	57 (2.6%)	6 (7.0%)	25 (2.6%)	18 (2.0%)	1 (0.6%)	7 (9.3%)	
Incidentally detected	1446 (66.3%)	20 (23.3%)	611 (62.8%)	646 (73.1%)	114 (69.9%)	55 (73.3%)	
Torsion, *n* (%)	32 (1.5%)	6 (7.0%)	9 (0.9%)	11 (1.2%)	5 (3.1%)	1 (1.3%)	0.000 ^a^
Tumour size (cm)	7.5 ± 3.7 (6.6, 0.8–30.0)	8.6 ± 4.2 (8.0, 2.0–20.4)	8.0 ± 3.9 (7.0, 0.8–30.0)	7.2 ± 3.4 (6.4, 1.0–22.4)	6.3 ± 2.5 (6.0, 1.0–18.9)	6.9 ± 4.3 (5.8, 1.9–27.0)	0.000 ^b^
Bilaterality, *n* (%)	321 (14.7%)	13 (15.1%)	176 (18.1%)	120 (13.6%)	8 (4.9%)	4 (5.3%)	0.000 ^a^
Serum CA125 (U/mL) *	26.2 ± 41.6 (17.0, 1–774) n = 1686	25.7 ± 29.1 (18.9, 5–232) n = 71	27.4 ± 41.6 (17.0, 2–608) n = 764	23.9 ± 29.6 (17.5, 1–371) n = 673	29.7 ± 75.7 (16.25, 2–774) n = 122	31.3 ± 67.2 (14.35, 5–470) n = 56	0.026 ^b^
Serum CA125 > 35 U/mL	226 (13.4%)	8 (11.3%)	119 (15.6%)	78 (11.6%)	11 (9.0%)	10 (17.9%)	0.154 ^a^
Serum CA19-9 (U/mL) *	72.5 ± 271.5 (20.9, 0–4634) n = 827	183.7 ± 435.3 (38.9, 1–2291) n = 37	76.2 ± 265.5 (23.6, 0–4634) n = 406	60.5 ± 264.4 (18.5, 1–4368) n = 302	22.8 ± 28.9 (13.5, 1–138) n = 50	87.8 ± 350.2 (12.2, 1–1997) n = 32	0.000 ^b^
Serum CA19-9 > 27 U/mL	347 (42.0%)	24 (64.9%)	187 (46.1%)	115 (38.1%)	12 (24.0%)	9 (28.1%)	0.000 ^a^
Preoperative CT or MRI	591 (27.1%)	39 (45.3%)	269 (27.6%)	213 (24.1%)	35 (21.5%)	35 (46.7%)	0.000 ^a^
Intraoperative frozen biopsy	116 (5.3%)	4 (4.7%)	51 (5.2%)	32 (3.6%)	12 (7.4%)	17 (22.7%)	0.000 ^a^
Follow-up duration (months)	24.0 ± 31.0 (10, 0–152)	36.5 ± 39.2 (25.5, 0–146)	26.3 ± 32.4 (12, 0–152)	21.6 ± 28.9 (9, 0–148)	20.7 ± 28.3 (9, 0–131)	15.8 ± 26.5 (3, 0–114)	0.000 ^b^

^a^ Chi-square test; ^b^ Kruskal–Wallis test, n, number; mens, menstruation; CA125, cancer antigen 125; CA19-9, cancer antigen 19-9, CT, computed tomography; MRI, magnetic resonance image, * normal tumour marker level: CA125 0-35 (U/mL), CA 19-9 0-27 (U/mL) in our institution.

**Table 2:** Number of patients with benign ovarian tumour associated with ovarian teratoma, according to age group.

Age Group	All	Group 1 (<20 Years)	Group 2 (20–30 Years)	Group 3 (30–40 Years)	Group 4 (40–50 Years)	Group 5 (≥50 Years)
Number of patients	2152	83	959	873	162	74
Benign ovarian tumour, *n* (%)	104 (4.8%)	1 (1.2%)	44 (4.6%)	41 (4.7%)	10 (6.2%)	8 (10.8%)
Ovarian endometrioma	76	0	35	32	6	3
Mucinous cystadenoma	21	1	7	7	3	3
Serous cystadenoma	3	0	1	1	0	1
Brenner tumour	1	0	0	0	0	1
Mucinous adenofibroma	1	0	0	1	0	0
Serous cystadenofibroma	1	0	0	0	1	0
Fibroma	1	0	1	0	0	0

**Table 3:** Number of patients with malignant ovarian tumour associated with ovarian teratoma, according to age group.

Age Group	All	Group 1 (<20 Years)	Group 2 (20–30 Years)	Group 3 (30–40 Years)	Group 4 (40–50 Years)	Group 5 (≥50 Years)	*p*-Value
Number of patients Intraoperative frozen biopsy	2181 116 (5.3%)	86 4 (4.7%)	973 51 (5.2%)	884 32 (3.6%)	163 12 (7.4%)	75 17 (22.7%)	
Malignancy, *n* (%)	29 (1.3%)	3 (3.5%)	13 (1.3%)	11 (1.2%)	1 (0.6%)	1 (1.3%)	0.442
Immature teratoma	11 (0.5%)	1	8	2	0	0	
Associated ovarian malignancy	9 (0.4%)	2	3	4	0	0	
Malignant transformation	9 (0.4%)	0	2	5	1	1	
Malignancy was confirmed on frozen biopsy	12 (41.4%)	2 (66.7%)	6 (46.2%)	4 (36.4%)	0 (0%)	1 (100%)	

**Table 4:** Comparison of MCT vs. malignant ovarian tumours associated with ovarian teratoma.

Tumour Group	Group A: MCT (n = 2152)	Group B: Immature Teratoma (n = 11)	Group C: Combined Malignancy (n = 9)	Group D: Malignant Transformation (n = 9)	A vs. B	A vs. C	A vs. D
Age (years)	30.9 ± 8.1 (30.0, 7–82)	24.9 ± 5.3 (24.0, 15–35)	27.0 ± 8.1 (27.0, 16–38)	37.1 ± 12.6 (33.0, 28–69)	0.005	0.261	0.046
Initial symptom, *n* (%)					<0.001	0.375	0.335
Irregular mens	105 (4.9%)	0	1 (11.1%)	0			
Abnormal bleeding	139 (6.5%)	0	0	0			
Abdominal pain	429 (19.9%)	2 (18.2%)	2 (22.2%)	0			
Palpable mass or abdominal distension	52 (2.4%)	4 (36.3%)	1 (11.1%)	0			
Incidentally detected	1427 (66.3%)	5 (45.5%)	5 (55.6%)	9 (100%)			
Torsion, *n* (%)	31 (1.4%)	1 (9.1%)	1 (11.1%)	0	0.152	0.126	1.0
Tumour size (cm)	7.5 ± 3.6 (6.6, 0.8–30.0)	16.0 ± 4.3 (16.0, 10.0–25.0)	9.0 ± 5.2 (7.0, 2.0–18.0)	9.0 ± 4.9 (7.0, 4.0–18.0)	<0.001	0.416	0.419
Serum CA125 (U/mL)	25.4 ± 39.2 (17.0, 1–774) (n = 1661)	119.7 ± 122.3 (35.0, 12–325) (n = 11)	67.5 ± 74.2 (24.1, 15–180) (n = 6)	50.7 ± 102.1 (13.1, 9–303) (n = 8)	0.002	0.066	0.373
Serum CA19-9 (U/mL)	66.1 ± 220.5 (21.0, 0–4368) (n = 808)	723.7 ± 1610.3 (43.3, 4–4634) (n = 8)	64.5 ± 63.0 (52.7, 7–152) (n = 6)	71.8 ± 139.7 (13.8, 3–322) (n = 5)	0.306	0.547	0.428
Preoperative CT or MRI	573 (12.6%)	11 (100%)	4 (44.4%)	3 (33.3%)	<0.001	0.259	0.707
Suspicious malignancy on CT or MRI finding	39 (6.8%)	4 (36.4%)	1 (25%)	0 (0%)	<0.001	0.141	1.0
Intraoperative frozen biopsy	100 (4.6%)	8 (72.7%)	7 (77.8%)	1 (11.1%)			
Malignancy was confirmed on frozen biopsy	0 (0%)	5 (62.5%)	6 (85.7%)	1 (100%)			
Follow-up duration (months)	23.8 ± 30.9 (10.0, 0–152)	43.2 ± 36.0 (43.0, 0–117)	40.7 ± 39.0 (29.0, 0–105)	39.4 ± 29.6 (39.0, 0–79)			

## Data Availability

The raw data supporting the conclusions of this article will be made available by the authors on request.

## Supplementary Materials

The following supporting information can be downloaded at: https://www.mdpi.com/article/10.3390/jcm14165872/s1, Table S1: Characteristics of malignancy associated cystic teratoma in our institution.
