# Supplementary material for: Age-Specific Characteristics and Malignancy Risk of Ovarian Teratomas: A Retrospective Single-Centre Study
Source: J Clin Med. 2025 Aug 20;14(16):5872. doi: 10.3390/jcm14165872 (PMC12387111; doi:10.3390/jcm14165872)
Supplement: Supplementary file 1 [file jcm-14-05872-s001.zip › jcm-3766819-supplementary.pdf]

Supplementary Table S1. Characteristics of malignancy associated cystic teratoma in our institution (*n*=29).

	Age (yrs)	Marital status	P	Symptoms at diagnosis	Preop CT or MRI	Elevated tumor marker	Tumor size (cm)	Frozen	Extraovarian disease (size, cm), LN	Operation name	Pathology	FIGO stage	Adjuvant therapy	F/U after operation (months)
1	15	Virgin	0	Abdominal distension	CT<br>CT	CA19-9 39	18.2	o<br>o	Omentum& Peritoneum: gliomatosis peritonei<br>Cul-de-sac, peritoneum, omentum: Gliomatosis peritonei(multiple)	LSC-RSO, omental mass& peritoneum resection<br>2nd look OP LSC-peritoneal masses resection, TO	Immature teratoma, high grade (grade2)<br>Gliomatosis peritonei	IA G2	BEP x3	60 Mo
2	21	Virgin	0	Abdominal pain (d/t torsion)	CT	CA19-9 47.6	8.1 (Rt 5.5)	x	x<br>x	LSC-BOCE<br>LSC-peritoneal resection, PO, RO wedge resection	LO: Immature teratoma grade 1 RO: MCT + torsion<br>RO : Remnant MCT Peritoneum&omentum : free	IC1 G1	x	51Mo
3	22	Virgin	0	Palpable mass	CT	CA125 114.5 AFP 21.2	17.0	o	x	Explo-RSO, Appe, Rt. PALN sampling	Immature teratoma, grade 2 Paraaortic : free	IA G2	BEP x5	117Mo
4	22	Virgin	0	Palpable mass	CT<br>x		16.0	x<br>x	x<br>x	SPA-ROCE<br>TPA-RSO, Lt. ovary mass excision, TO, Rt. Pelvic peritoneactomy	RO immature teratoma, grade 2<br>RO : without residual tumor LO : fibroma Omentum&peritoneum : free	IC1 G2	BEP x3	3Mo, f/u loss after BEP 3cycle
5	24	Virgin	0	Palpable mass	MRI	CA125 265.4 CA19-9 918.5	15.0 (Rt 10)	o	x	Explo-BOCE	LO: Immature teratoma, grade 2 (focal primitive neuroepithelial element. maximal dimension: 1.5x1 cm) RO: MCT	IC1	x	38Mo
6	24	Virgin	0	Abdominal pain	CT	CA125 262.9 ROMA:15.4	20	o	x	SPA-LSO	LO: Immature teratoma, grade 2.	IC1 G2	BEP x3	43Mo
7	26	Single	0	Incidentally detected	CT	CA125 325.3 CA19-9 4634.3 AFP 1366.0 CEA 2.98	17	o	x	Explo-RSO, PO, LO & peritoneum Bx	RO: Immature teratoma, grade 3. LO & omentum & peritoneum : free	IA G3	BEP x4	79Mo
8	26	Single	0	Abdominal pain	CT	CA125 211.8 CA19-9 130.7	20 (Lt 5)	o	x	Explo-RSO, LOCE, condyloma excision	RO: Immature teratoma, grade 3/3 LO: MCT Vulva,excision: condyloma acuminatum	IA G3	BEP x2	1Mo, f/u loss after BEP 2cycle
9	28	Married	0	Incidentally detected	CT	-	5.0 (Rt 4.5)	o	x<br>x	LSC-BOCE<br>SPA-LSO, TO, RO&peritoneum Bx	LO: Immature teratoma, grade I RO: MCT<br>LSO : no residual tumor RO&Omentum&Peritoneum : Free	IC1 G1	x	24Mo Subsequent pregnancy: Full term delivery at postop 13Mo
10	31	Married	0	Incidentally detected	CT	-	9.9	x	x	LSC-LOCE, myomectomy	LO: Immature teratoma, grade 1 Myomectomy : myoma	IC1 G1	x	59Mo
11	35	Married	2	Incidentally detected	CT	-	13.6	o	x	SPA-LSO	LO: Immature teratoma, grade 2/3	IA G2	x	0Mo Transfer to other hospital
12	16	Virgin	0	Abdominal pain	x	-	10.8 (Rt 6.7)	x	x	LSC-BOCE, detorsion	LO:Mucinous borderline RO: MCT + torsion	-	x	2Mo Transfer to other hospital d/t recurrent BO cyst since postop 1Mo
13	18	Virgin	0	Irregular menstruation	CT	-	5.7 (Rt 1.9)	o	x	SPA-BOCE, TO, Appe,	LO:mucinous carcinoma (FIGO	IIB	TC x6	29Mo Suspicious of

					x			x	Pelvic peritoneum 1.5cm, 0.7cm	TLH, BSO, pelvic perineactomy, BPLND, BPALND	2) with pseudomyxoma ovarii<br>RO: MCT<br>Pelvic peritoneum : Presence of mucinous carcinoma			peritoneal seeding and malignant ascites, bladder metastasis or primary ca : transfer to other hospital
14	20	Single	0	Abdominal pain	x	CA125 144.2<br>CA19-9 151.7	25.0<br>(Lt 5)	o	x	LSC-RSO, LOCE, TO, Appe	LO: MCT<br>RO: Mucinous cystadenocarcinoma, well differentiated, FIGO I (spontaneous rupture)	IC2	TC x6	105Mo
15	23	Single	0	Incidentally detected	CT	CA19-9 93.5	9.8<br>(Lt 4.2)	o	x	LSC-BOCE	LO: mucinous cystadenoma + MCT<br>RO:mucinous borderline + MCT	-	x	0Mo<br>transfer to other hospital
16	27	Single	0	Constipation	CT	CA125 179.8<br>AFP 23340	9.2	o	x	RSO, rectal serosa mass excision, PO	RO: yolk sac tumor c capsular extension + MCT	IIB	BEPx4<br>2nd line POMBx1	7Mo<br>Elevated AFP after BEP 4cycle-> 2nd line POMB,<br>ARF complicated with CTx: transfer to other hospital
17	32	Single	0	Incidentally detected	x	-	7.3	x		LSC-LOCE, myomectomy	LO: mucinous borderline + focal MCT	-	x	84Mo
18	34	Married	0	Incidentally detected	x	CA19-9 112.7	6.7	o	x	LSC-LOCE, myomectomy, RO drilling	LO: cellular fibroma + MCT	-		26Mo<br>Subsequent pregnancy: ANC at other hospital
19	35	Married	2	Incidentally detected	MRI	-	10.7<br>(Rt 5.2)	o	x	LAVH, BSO, TO, Appe	LO: mucinous borderline<br>RO: MCT	-	x	79Mo
20	39	Married	1	Growing ovarian cyst during pregnancy	x	-	13.5	o: benign	x	R C/S+ROCE	RO: mucinous cystic tumor of borderline malignancy+MCT	-	x	1Mo<br>Cooperation with R C/S, transfer to other hospital
21	28	Married	0	Incidentally detected	CT	-	4.0	x	x<br>x	SPA-LOCE<br>SPA-LSO, adhesiolysis	LO: papillary thyroid carcinoma arising in mature cystic teratoma<br>*Size of papillary thyroid carcinoma : about 0.7 cm<br>LSO : Free from tumor	-	x	76Mo<br>Subsequent pregnancy at postop 13Mo:<br>IVF-ET, full-term twin C/S
22	29	Single	0	Incidentally detected	x	-	5.0	x	x	LSC-ROCE	RO: MCT showing thyroid tissue and focal carcinoid tumor 0.2cm	-		39Mo
23	30	Single	0	Incidentally detected	x	CA125 30.9<br>CA19-9 321.5	6.1	x	x	LSC-LOCE	Lt MCT + strumal carcinoid<br>*size of carcinoid tumor : about 2 mm)	-	x	39Mo<br>Subsequent pregnancy: natural preg, full term NSVD at postop 37Mo
24	32	Single	0	Incidentally detected	x	-	5.9	x	x	SPA-LOCE	MCT associated with strumal carcinoid.<br>*Size of strumal carcinoid : 0.3 cm	-	x	46Mo<br>Subsequent pregnancy: IVF-ET, full term Br C/S at postop 45Mo
25	33	Single	0	Incidentally detected	x	CA125 302.8<br>SCC 5.6	10.2	o	Appendix & omentum : free	LSC-LSO, infracolic omentectomy, Appe	LO:<br>INVASIVE SQUAMOUS CELL CARCINOMA, moderately differentiated, arising from MCT with extension to the capsule	IC2 (capsular involvement)	IP CTx (Cisplatin) TC x6	79Mo<br>Subsequent pregnancy: natural pregnancy, full-term FD C/S at postop 21Mo
26	36	Married	0	Incidentally detected	MRI	-	6.2	x	At other hospital, on EGD finding-well differentiated neuroendocrine tumor	LSC-LOCE, myomectomy	LO: carcinoid tumor, mixed insular and trabecular type	-	x	0Mo<br>Transfer to other hospital
27	37	Married	0	Infertility	MRI	-	12.5	x	x	LSC-BOCE	LO: Strumal	-	x	16Mo

							(Rt 3.4)				carcinoid, associated with mature cystic teratoma.<br>*Size of strumal carcinoid : 0.5 cm			Subsequent pregnancy: IVF ET, twin C/S at postop 14Mo
28	40	Single	0	Incidentally detected	x	x	6.8	x	x	LSC-LSO, myomectomy, paratubal cystectomy,	RO: MCT<br>LO: strumal carcinoid<br>(Size of strumal carcinoid : 0.4 cm)	-	x	60Mo
29	69	Married (menopause)	4	Incidentally detected	CT	-	18.0	o	x	LSC-LSO	LO: strumal carcinoid associated c mucinous cystadenoma, intestinal type	-	x	0Mo<br>Transfer to other hospital

Yrs, years; P, parity; Preop, preoperative; CT, computed tomography; MRI, magnetic resonance image; LN, lymph node; F/U, follow-up; CA19-9, Cancer antigen 19-9; LSC, laparoscopic; RSO, right salpingooophorectomy; B, Bleomycin; E, etoposide; P, cisplatin; Mo, months; OP, operation; TO, total omentectomy; d/t, due to; BOCE, bilateral ovarian cyst enucleation; LO, Left ovary; RO, Right ovary; MCT, mature cystic teratoma; PO, partial omentectomy; free, free from tumor; CA125, Cancer antigen 125; AFP, alpha-fetoprotein; Explo, exploratory; Appe, appendectomy; Rt, Right; PALN, paraaortic lymph node; SPA, single port access; ROCE, right ovarian cyst enucleation; TPA, two port access; Lt, left; ROMA, risk of ovarian malignancy algorithm; LSO, left salpingooophorectomy; CEA, carcinoembryonic antigen; LOCE, Left ovarian cyst enucleation; postop, postoperative; BO, both ovary; T, paclitaxel; C, carboplatin; POMB, cisplatin+vincristine+methotrexate+bleomycin; ARF, acute renal failure; CTx, chemotherapy; ANC, antenatal care; LAVH, laparoscopic assisted vaginal hysterectomy; R C/S, repeat cesarean section; IVF-ET, in vitro fertilization – embryo transfer; C/S, cesarean section; NSVD, normal spontaneous vaginal delivery; Br, Breech; SCC, squamous cell carcinoma related antigen; FD, fetal distress; EGD, esophagogastroduodenoscopy. *Elevated tumor marker : CA125 >35 (U/mL), CA 19-9 >27 (U/mL), AFP>8.1 (ng/mL), CEA>5.0 (ng/mL), SCC>1.5 (ng/mL).
